# Supplementary material for: mRNA targeting eliminates the need for the signal recognition particle during membrane protein insertion in bacteria
Source: Cell Rep. 2023 Feb 25;42(3):112140. doi: 10.1016/j.celrep.2023.112140 (PMC10066597; doi:10.1016/j.celrep.2023.112140)
Supplement: Document S1. Figures S1–S7 and Tables S1–S4 [file mmc1.pdf]

Cell Reports, Volume *42*

Supplemental information

mRNA targeting eliminates the need for the signal recognition particle during membrane protein insertion in bacteria

Pinku Sarmah, Wenkang Shang, Andrea Origi, Mariya Licheva, Claudine Kraft, Maximilian Ulbrich, Elisabeth Lichtenberg, Annegret Wilde, and Hans-Georg Koch

Supplemental Information Titles and Legends

Legends to Supplementary Figures

Figure S1: Fluorescence distribution profile of *E. coli* cells expressing different mRNAs. **(**Related to Figure 1). **(A)** *E. coli* cells producing either MS2-Venus or MS2-Venus together with the indicated mRNAs were grown as described in the legend to Fig. 1. Subsequently, fluorescence distribution of the Nile red fluorescence (blue) and the MS2-Venus fluorescence (orange) was analyzed in individual cells and is displayed as relative fluorescence intensity. Polar areas of cells were excluded and quantification was performed along a vertical axis in mid-cell as indicated in the micrograph. The Jensen-Shannon divergence (JSD) value of the analyzed cell is indicated and JSD values close to "0" indicate almost identical distributions of Nile red and MS2-venus fluorescence. The averaged JSD values of multiple cells (n > 60-80) are shown in Fig. 1. **(B)** *In vivo* localization of *yohP* mRNA in the presence and absence of the 5`- and 3`- UTRs. Cells just expressing MS2-Venus were used as a control. Sample preparation and imaging was performed as described in Fig.1. **(C)** Jensen-Shannon divergence of the samples in (B). Statistical analyses were performed with the Satterthwaite corrected unpaired two-sided Student t-test, using cells expressing just MS2-venus without mRNA as a reference. (***) refers to p-values ≤0.001. (**D**) RNA-FISH of wild-type *E. coli* cells using the TAMRA-labelled *yohP* probes as described in Fig, 1C. Note that the expression level of the chromosomal *yohP* transcript was too low to be clearly localized by microscopy. As negative controls, the experiment was performed with TAMRA-labelled *psbA* probes, specific for the *psbA* mRNA which encodes a photosystem II protein from the cyanobacterium *Synechocystis* sp PCC6803 and without adding probes. **(E)** Quantification of the RNA-FISH experiments as in (D) in cells expressing the *yohP* mRNA with (red line) and without the 6x MS2 tag (black line). FISH-images were quantified by monitoring the mRNA localization profiles perpendicular to the long

axis of the cells. The standard deviations of analyzing 33 cells expressing *yohP*-MS2 mRNA and 32 cells expressing *yohP* mRNA are shown in brown and grey, respectively.

Figure S2: Nucleotide sequences and predicted secondary structures of the *yohP* mRNAs used in this study. (Related to Figures 1, 2, and 3). **(A)** *yohP* mRNA variants with different nucleotide composition were designed without changes in the predicted translation product. Nucleotides that were replaced are indicated in red. U-rich*, C-rich*, A-rich* and G-rich* refer to *yohP* variants with changes in the nucleotide composition that have only a small impact on the predicted secondary structures of the mRNA. *yohP*(Δx-x) refer to *yohP* variants with loop deletions and *yohP*(x-x subs) to variants with substituted nucleotides in the mRNA loops. **(B)** Nucleotide content and length of the different *yohP* variants shown in (A). **(C) – (E)** Predicted secondary structures of the indicated mRNAs. Structures were predicted by using the *RNAfold* webserver (http://rna.tbi.univie.ac.at/ cgi-bin/RNAWebSuite/RNAfold.cgi). All variants were generated by Q5 site-Directed-Mutagenesis kit and NEBuilder HiFi Assembly Master Mix.

Figure S3: Influence of nucleotide composition and secondary structure on membrane binding of *yohP* mRNAs. (Related to Figures 2 and 4). **(A)** Jenson-Shannon divergence (JSD) plot of *yohP* mRNAs with modified nucleotide composition as shown in Fig. 2 and listed in Fig. S2. The JSD was evaluated with scores between 0 (identical distribution) and 1 (represents maximally different distribution) and is based on scoring 60-80 individual cells. Individual cells are shown in Figs. 2. Statistical analyses were performed with the Satterthwaite corrected unpaired two-sided Student t-test, using cells expressing wild type *yohP* mRNA together with MS2-venus as reference. (*) refers to *p*-values ≤0.05 and (**) to *p*-values ≤0.01. **(B)** Total RNA isolated from cells expressing different mRNAs and separated by agarose gel electrophoresis. 2 µg total RNA were loaded and the gel was stained with Sybr green. **(C)** & **(D)** Northern-blot analyses of different *yohP* mRNAs. The total RNA (2 µg) shown in B was blotted onto a nylon membrane and detected with a ^{32}P-labelled oligonucleotide,

complementary to the MS2-stem loop. **(E)** & **(F)** Quantifications of three independent Northern blot experiments as shown in (C) and (D) were performed by using *Image J* and the mean values and the SEM values are indicated. **(G)** Jenson-Shannon divergence (JSD) plot of *yohP* mRNAs with loop deletions as shown in Fig. 3 and listed in Fig. S2. Statistical analyses were performed as in (A).

Figure S4: The *yohP* mRNAs used for imaging are not translated and bind to liposomes of different lipid composition. (Related to Figures 4 and 5). **(A)** *In vitro* translation of *yohP* mRNAs either containing the ribosome-binding site (+RBS) or lacking it (-RBS). The *in vitro* translation system consisted of a purified cytosolic extract, purified ribosomes and an amino acid mixture containing ^{35}S-labelled methionine and cysteine. Samples were separated by SDS-PAGE and analyzed by autoradiography. YohP tends to dimerize and the YohP monomer and dimer are indicated. **(B)** The *yohP* mRNA was *in vitro* transcribed and ^{32}P labeled. After purification, the mRNA was incubated with liposomes (2.5 µg/µl) of different lipid composition (PE, phosphatidyl-ethanolamine, PC, phosphatidyl-choline; CL; cardiolipin; PG, phosphatidyl-glycerol). After incubation, liposomes and the bound mRNA were pelleted by centrifugation and the membrane fraction (P) and the soluble fraction (S) were separated on a urea-PAGE-gel and analyzed by autoradiography. **(C)** Quantification of the data shown in (B). Shown are the mean values of at least three experiments and SEM in indicated by error bars. Statistical analyses were performed with the Satterthwaite corrected unpaired two-sided Student t-test, using the values after incubation without INVs as reference. (*) refers to *p*-values $\leq$0.05; (**) to *p*-values $\leq$0.01, and (***) to *p*-values $\leq$0.001. **(D)** Immune detection of SecY in different INVs. SecYEG-OE refers to INVs from a SecYEG-overproducing strain and SecY(ΔC4-6)EG to INVs from an *E. coli* strain that overproduces the truncated SecYEG complex. Antibodies against YidC were used as loading control. **(E)** Cartoon showing the TM-MS2-venus construct

(left panel) and a microscopic image showing *E. coli* cells producing TM2-MS2-venus (right panel). The cartoon was generated using Biorender (https://biorender.com/).

Figure S5: Localization of different SecY-variants in the *E. coli* membrane. (Related to Figures 4 and 5). **(A)** Different variants of the SecYEG translocon with a C-terminally YFP-tagged SecY were produced in *E. coli* and membrane localization was analyzed by fluorescence microscopy. ΔC4, ΔC5, Δ6 and ΔC4-6 refer to SecY deletions that lack either the cytosolic loop C4, C5, C6 or all three loops, respectively. The scale bar refers to 2.5 µm. (**B**) Immune detection of YidC in wild type INVs and INVs from a YidC overproducing strain. The small size shift is due to the presence of a His-tag in the plasmid- encoded YidC. **(C)** *In vitro* binding of uracil-rich and cytosine-rich *yohP* mRNAs to the indicated INVs was performed as described in the legend to Fig. 4 and a quantification is shown in Fig. 4E. **(D)** *In vitro* translation of *yohP* variants that either contain or lack the MS2 stem loop. *In vitro* translation was performed as described in Fig. S4 in the presence of wild type INV and one half of the reaction was treated with proteinase K for detecting membrane inserted YohP.

Figure S6: mRNA targeting is translation independent. (Related to Figures 1-3). **(A-D)** For validating that mRNA targeting occurs independently of translation, mRNA targeting was followed in the absence or presence of antibiotics. Kasugamycin (0.1 mg/ml) or Puromycin (0.2 mg/ml) were present during the last 10 min of MS2-Venus induction. Microscopy was performed as described in the legend to Fig. 1. The scale bar refers to 2.5 µm. **(E)** JSD-plot of 60-80 individual cells, treated as in (A-D). Statistical analyses were performed with the Satterthwaite corrected unpaired two-sided Student t-test, using cells expressing the corresponding mRNA in the absence of antibiotics as reference. (*) refers to *p*-values ≤ 0.05 and (n.s.) to not significant.

Figure S7: Validation of the experimental strategy for determining the role of SRP in membrane protein insertion. (Related to Figures 5 and 6)**. (A)** Urea treatment removes large amounts of FtsY and SRP (Ffh) from INVs. Urea-treated INVs (U-INVs) were generated by treating INVs with 6 M urea; after incubation and centrifugation, U-INV (2.5 µg) were separated by SDS-PAGE and after western-blotting detected with antibodies against Ffh, the protein component of the bacterial SRP, and FtsY, the bacterial SRP receptor. Untreated INVs served as control. **(B)** Quantification of the pulse-chase experiment shown in Fig. 6A. Quantification of three independent experiments was performed via *Imagequant*. For quantification, the radioactive signal in the pellet fraction was divided by the sum of the radioactive signals in the pellet and soluble fraction and is displayed as % YohP insertion. Shown are the mean values and the standard errors of the mean (SEM), which were calculated via *GraphpadPrism*. **(C)** Pulse-chase experiment for monitoring the fractionation procedure. The cytosolic protein YchF served as a control to ensure that the procedure described in the legend to Fig.6 allows to separate soluble from inner membrane proteins. Cells were grown on minimal media and YchF production was induced by the addition of IPTG. ^{35}S-labelled methionine/cysteine mix was then added, followed by 5 min incubation. Cells were chased for 1, 5 and 10 min with an excess of non-radioactive methionine and cysteine. Cells were then lysed by sonication as described in the legend to Fig. 6 and separated by ultracentrifugation in the soluble fraction and the membrane fraction. Both fractions were then separated on SDS-PAGE and the gel was analyzed by autoradiography. The position of 40 kDa YchF is indicated. **(D)** Norvaline inhibits global translation in *E. coli*. Cells were grown on minimal media and treated for 90 min with norvaline when indicated. ^{35}S-labelled methionine/cysteine mix was then added, followed by 5 min incubation. Cells were chased for 1, 5 and 10 min with an excess of non-radioactive methionine and cysteine. After separation on SDS-PAGE, the gel was analyzed by autoradiography. YohP is indicated.

Table S1: *E. coli* strains used in this study

Name	Description	Application	Reference
DH5α	*supE44 ΔlacU169 (Φ80lacZΔM15)hs dR17 recA1 endA1 gyrA96 thi-1 relA1*	Plasmid isolation and storage	[1]
BL21	*E. coli B F- dcm ompT hsdS(rBmB) gal [malB^{+}] K-12(λS)*	Protein expression *in vivo*, protein purification	Merck, Darmstadt, Germany
C43(DE3)	F – *ompT hsdSB (rB- mB-) gal dcm* (DE3)	Protein expression *in vivo*, protein purification	[2]

Table S2: Plasmids used in this study

Plasmids used for *in vivo* mRNA imaging lacked the Shine-Dalgarno sequence (SD).

**Name**	**Resistance/ Description**	**Application**	**Reference**
pBad24	Amp	Cloning vector	[3]
pET-19b	Amp	Cloning vector	Merck, Darmstadt, Germany
pRS1	Amp	Cloning vector	[4]
pRS1-YohP-MS2.6x	Amp	In vivo and in vitro expression YohP	[5]
pBad24-YohP$_{His6}$,	Amp	In vivo expression YohP	[5]
pBad22-SecE(Y$_{GFP}$)G	Amp	In vivo expression	[6]
pBad22-SecE(YΔC4-C6$_{GFP}$)G	Amp	In vivo expression	This study
pBad22-SecE(YΔC4$_{GFP}$)G	Amp	In vivo expression	This study
pBad22-SecE(YΔC5$_{GFP}$)G	Amp	In vivo expression	This study
pBad22-SecE(YΔC6$_{GFP}$)G	Amp	In vivo expression	This study
pTRc99a-SecY$_{His}$EG	Amp	INV	[7]
pBad22-SecE(YΔC4-C6)G	Amp	INV	This study
pBad22-SecE(YΔC4)G	Amp	INV	This study
pBad22-SecE(YΔC5)G	Amp	INV	This study
pBad22-SecE(YΔC6)G	Amp	INV	This study
pTRC99a-YidC	Amp	INV	[8]
pTRC99a-FtsY	Amp	Protein purification	[9]
pTRC99a-Ffh	Amp	Protein purification	[10]
pT7/3a	Amp	In vitro transcription 4.5S RNA	[11]
pBad-MS2-venus	Amp	In vivo expression MS2-venus	This study
pSC.YohP-MS2.6x	Cm	In vivo expression of *yohP* mRNA	[5]
pSC.BglB-MS2.6x	Cm	In vivo expression of *bglB* mRNA	[5]
pSC.SecY-MS2.6x	Cm	In vivo expression of *secY* mRNA	This study
pSC.YohP-MS2-U-rich.6x	Cm	In vivo expression of U-rich *yohP* mRNA	This study
pSC.YohP-MS2-C-rich.6x	Cm	In vivo expression of C-rich *yohP* mRNA	This study
pSC.YohP-MS2-G-rich.6x	Cm	In vivo expression of G-rich *yohP* mRNA	This study
pSC.YohP-MS2-A-rich.6x	Cm	In vivo expression of A-rich *yohP* mRNA	This study
pSC.YohP(Δ4-30)-MS2.6x	Cm	In vivo expression of *yohP*(Δ4-30) mRNA	This study

pSC.YohP(Δ10-27)-MS2.6x	Cm	In vivo expression of *yohP*(Δ10-27) mRNA	This study
pSC.YohP(Δ46-60)-MS2.6x	Cm	In vivo expression of *yohP*(Δ46-60) mRNA	This study
pSC.YohP(4-30subst)-MS2.6x	Cm	In vivo expression of *yohP*(4-30subst) mRNA	This study
pSC.YohP(10-27subst)-MS2.6x	Cm	In vivo expression of *yohP*(10-27subst) mRNA	This study
pSC.YohP(46-60subst)-MS2.6x	Cm	In vivo expression of *yohP*(46-60subst) mRNA	This study
pBAD24.SecY-2TM_MS2-Venus	Amp	INV	This study
pRS1.YohP_MS2.6X	Amp	In vitro and in vivo study	This study
pRS1.YohP-MS2-U-rich.6x	Amp	In vitro and in vivo study	This study
pRS1.YohP-MS2-C-rich.6x	Amp	In vitro and in vivo study	This study
pRS1-YohP(ΔRBS).MS2.6X	Amp	In vitro protein synthesis	This study
pSc.YohP-MS2.6X-A-rich*	Cm	In vivo expression of *yohP* A-rich* mRNA	This study
pSc.YohP-MS2.6X-U-rich*	Cm	In vivo expression of *yohP* U-rich* mRNA	This study
pSc.YohP-MS2.6X-G-rich*	Cm	In vivo expression of *yohP* G-rich* mRNA	This study
pSc.YohP-MS2.6X-C-rich*	Cm	In vivo expression of *yohP* C-rich* mRNA	This study
pSc.YohP.5’-3’ UTR.MS2.6X	Cm	In vivo expression of *yohP* mRNA with 5’-3’-UTR	This study
pET19b-YchF	Amp	In vivo protein synthesis	[12]

Table S3: Oligonucleotides used in this study

Name	Sequence 5'-3'	Application
C4_trunc_fw	CATTTACCGCTGAAAGTGAATAGGC	Deletion of C4 loop SecY
C4_trunc_rev	GCGGCGTTGACCACGCTCAAC	Deletion of C4 loop SecY
C5_trunc_fw	AAAGTAATGACCCGCCTGACCCT	Deletion of C5 loop SecY
C5_trunc_rev	CTTTGCTGTTTCACGCGGGTTGAA	Deletion of C5 loop SecY
C6_trunc_fw	TTAGCGGCGGCGGGATCCGGA	Deletion of C6 loop SecY
C6_trunc_rev	GGACATCATCAGAGTTTGCACTTGA	Deletion of C6 loop SecY
pSc_Opening_Fw	GATGATATTTTAAGTAATTATAAC	Linearization of the plasmid pSc for Gibson assembly
pSc_Opening_rev	CTCAGGTCGACGTCCCATGGCCATTCG AAT	Linearization of the plasmid pSc for Gibson assembly
U-rich_fw	AAT TCA GGA GGT CGA CCT GAG ATG AAA ATT ATT CTT TGG GCT GTT TTG ATT ATT TTC CTT ATT GGT CTTC TTG TTG TTA CTG GTG TTT TTA AGA TGA TTT TTT AAG	U-rich variant of YohP
U-rich_rev	AATTCAGGAGGTCGACCTGAGATGAA AA TCATACTCTGGGCCGTACTGATCATC TTC CTGATCGGGCTACTGGTGGTGA CCGGCG TATTCAAGATGATATTCT AAG	U-rich variant of YohP
C-rich_fw	AATTCAGGAGGTCGACCTGAGATGAAAAT CATACTCTGGGCCGTACTGATCATCTTCCT GATCGGGCTACTGGTGGTGACCGGCGTAT TCAAGATGATATTCTAAG	C-rich variant of YohP

C-rich_rev	GATCCTTAGAATATCATCTTGAATACGCCGGT CACCACCAGTAGCCCGATCAGGAAGATGATC AGTACGGCCCAGAGTATGATTTTCATCTCAG GTCGACCTCCTG	C-rich variant of YohP
G-rich_fw	GCC ATG GGA CGT CGA CCTG AGA TGA AGA TTA TAC TGT GGG CGG T	G-rich variant of YohP
G-rich-rev	ATA ATT ACT TAA AAT ATC ATC TTA AAA TAT CAT CTT AAA C	G-rich variant of YohP
A-rich_fw	CCA TGG GAC GTC GAC CTG AGA TGA AAA TAA TAC TAT GGG CAG TAT TAA TAA TAT TAG TAA CAG GAG TAT TTA AAA TGA TAT TTG ATG ATA TTT TAA GTA ATT A	A-rich variant of YohP
pRS1_Opening_Fw	TCGAGTAGCATAACCCCTTGGGGCCTCT AAACGGG	Linearization of pRS1 for Gibson assembly
pRS1_Opening_rev	CATGGGGTATATCTCCTTCTTAAAGTTA AACAAAATTATTT	Linearization of pRS1 for Gibson assembly
YohP_MS2-6X_Fw	GAAGGAGATATACCCCATGATGAAAAT TATACTCTGGG	In vitro translation and pulse chase
YohP_MS2-6X_rev	GGGGTTATGCTACTCGAGCTAGAACTAT AGCTAGCATG	In vitro translation and pulse chase
U-rich_MS2.6X_fw	TATACCCCATGAATGAAAATTATTCTTT GGGC	In vitro translation and pulse chase
C-rich_MS2-6X	AGAAGGAGATATACCCCATGAATGAAA ATCATACTCTGGGCCGTAC	In vitro translation and pulse chase
YohP (Δ4-30)_fw	ATTTTCCTGATTGGGCTACTGGTGG	Imaging study
YohP (Δ4-30)_rev	CATCTCAGGTCGACGTCCCATG	Imaging study
YohP (Δ10-27)_fw	ATTATTTTCCTGATTGGGCTACTGGTGGT	Imaging study
YohP (Δ10-27)_rev	AATTTTCATCTCAGGTCGACGTCC CA	Imaging study
YohP (Δ46-60)_fw	GGCGTATTTAAGATGATATTTTAA GATGATATTTTAAGTAATTATAACCC	Imaging study

YohP (Δ46-60)_rev	CCCAATCAGGAAAATAATCAA TACAGCCCAGAGTATAATT	Imaging study
YohP(4-30 subst)_Fw	GGGCAGTTTTAATCATTTTCCTGATTGG GCTACTGGTGGTGACTGGCGT	Imaging study
YohP(4-30 subst)_rev	ACAGAATGATCTTCATCTCAGGTC GACGTCCCATGGCCA	Imaging study
YohP(10-27 subst)_rev	ATTTTCATCTCAGGTCGACGTCCC ATGG CCATTCGA	Imaging study
YohP(10-27 subst)_fw	TACACACTCGGATGCATCGATTATTTTC CTGATTGGGCTACTGGT	Imaging study
YohP(46-60 subst)_fw	AGTCACAGGCGTATTTAAGATGATATTT TAAGGCGTATTTAAG	Imaging study
YohP(46-60 subst)_rev	ACGAGAAGCCCAATCAGG AAAATAATC	Imaging study
pBAD24 Opening_fw	GGAGTCTGGTCGTCGTAAGATGGCTTCT AACTTTACTCAGTTCG	Linearization of pBAD24 for Gibson assembly
pBAD24 Opening_rev	CTAATCCCGGTTGTTTAGCCATGG GTACCATGGTGAATTCCTC	Linearization of pBAD24 for Gibson assembly
SecY_2TM_fw	CAGGAGGAATTCACCATGGTACCCATG GCTAAACAACCGGGATTAG	INV preparation
SecY_2TM_rev	CGA ACT GAG TAA AGT TAG AAG CCA TCT TAC GAC GAC CAG ACT CC	INV preparation
A-rich*.6x_fw	CCA TGG GAC GTC GAC CTG AGA TGA AAA TTA TAC TCT GGG CTG TAT TAA TTA TTT TCC TAA TTG GGC TAC TGG TAG TAA CAG GCG TAT TTA AAA TGA TAT TTT AAG TAA TTA TAA CCC GGG CCC T	Imaging study
U-rich*.6x_fw	CCA TGG GAC GTC GAC CTG AGA TGA AAA TTA TAC TTT GGG CTG TAT TGA TTA TTT TTC TTA TTG GTC TAC TGG TGG TGA CTG GTG TTT TTA AGA TGA TTT TTT AAG TAA TTA TAA CCC GGG CCC T	Imaging study
G-rich*.6x_fw	CCA TGG GAC GTC GAC CTG AGA TGA AAA TTA TAC TGT GGG CGG TGT TGA TTA TTT TCC TGA TTG GGC TAC TGG TGG TGA CTG GCG TGT TTA AGA TGA TAT TTT AAG TAA TTA TAA CCC GGG CCC	Imaging study
C-rich*.6x_fw	CCA TGG GAC GTC GAC CTG AGA TGA AAA TCA TAC TCT GGG CTG TAT TGA TCA TCT TCC TGA TCG GCC TAC TGG TGG TGA CTG GCG TCT TCA AGA TGA TCT TCT AAG TAA TTA TAA CCC GGG CCC T	Imaging study

YohP-5'-3' UTR-MS2.6X_fw	GCC ATG GGA CGT CGA CCT GAG TAT ACA CTA AGT GAA TGA TAT CTT C	Imaging study
YohP-5'-3' UTR-MS2.6X_rev	TAG GGC CCG GGT TAT AAT TAC AGC CCG GGG GTG CAG GGG GCG	Imaging study

Table S4: *yohP* probes used in this study

Name	Sequence 5'-3'	Application
1	TCATTCACTTAGTGTATA	FISH
2	AAGATAAATCGGAAGATA	FISH
3	CGTTATCCATAAACGATT	FISH
4	AAAAACGAAGCCCTTTGC	FISH
5	TGCTGAATAAGTATAGGA	FISH
6	CGTTCCTTTATTTGTGAG	FISH
7	GAGTATAATTTTCATTGG	FISH
8	AATAATCAATACAGCCCA	FISH
9	CAGTAGCCCAATCAGGAA	FISH
10	AAATACGCCAGTCACCAC	FISH
11	ATTTTAAAATATCATCTT	FISH
12	ACCTGATGACATTAATTA	FISH
13	TATTCTCGTTATTTTCGG	FISH
14	GCAACAGGATGAGAGACT	FISH
15	AATGCACATGACAGGAGC	FISH
16	CCAGTGATTATATGAAGC	FISH
17	CCCTGCGCGCTCCTTGCG	FISH
18	CGGCGGCGATTGGCCGCC	FISH
19	GCCCGGGGGTGCAGGGGG	FISH
oligo probe for MS2	CTGCAGACATGGGTGATCCTCATGT	Northern blot

References

1. Hanahan, D. (1983) Studies on transfromation of Escherichia coli with plasmids, *Journal of molecular biology*. **166**, 557-580.

2. Miroux, B. & Walker, J. E. (1996) Over-production of proteins in Escherichia coli: mutant hosts that allow synthesis of some membrane proteins and globular proteins at high levels, *Journal of molecular biology*. **260**, 289-98.

3. Guzman, L. M., Belin, D., Carson, M. J. & Beckwith, J. (1995) Tight regulation, modulation, and high-level expression by vectors containing the arabinose PBAD promoter, *Journal of bacteriology*. **177**, 4121-30.

4. Jauss, B., Petriman, N. A., Drepper, F., Franz, L., Sachelaru, I., Welte, T., Steinberg, R., Warscheid, B. & Koch, H. G. (2019) Non-competitive binding of PpiD and YidC to the SecYEG translocon expands the global view on the SecYEG interactome in E. coli, *The Journal of biological chemistry*.
5. Steinberg, R., Origi, A., Natriashvili, A., Sarmah, P., Licheva, M., Walker, P. M., Kraft, C., High, S., Luirink, J., Shi, W. Q., Helmstädter, M., Ulbrich, M. H. & Koch, H. G. (2020) Posttranslational insertion of small membrane proteins by the bacterial signal recognition particle, *PLoS biology*. **18**, e3000874.
6. Kuhn, P., Weiche, B., Sturm, L., Sommer, E., Drepper, F., Warscheid, B., Sourjik, V. & Koch, H. G. (2011) The bacterial SRP receptor, SecA and the ribosome use overlapping binding sites on the SecY translocon, *Traffic (Copenhagen, Denmark)*. **12**, 563-78.
7. Collinson, I., Breyton, C., Duong, F., Tziatzios, C., Schubert, D., Or, E., Rapoport, T. & Kuhlbrandt, W. (2001) Projection structure and oligomeric properties of a bacterial core protein translocase, *The EMBO journal*. **20**, 2462-71.
8. Welte, T., Kudva, R., Kuhn, P., Sturm, L., Braig, D., Muller, M., Warscheid, B., Drepper, F. & Koch, H. G. (2012) Promiscuous targeting of polytopic membrane proteins to SecYEG or YidC by the Escherichia coli signal recognition particle, *Molecular biology of the cell*. **23**, 464-79.
9. Braig, D., Bar, C., Thumfart, J. O. & Koch, H. G. (2009) Two cooperating helices constitute the lipid-binding domain of the bacterial SRP receptor, *Journal of molecular biology*. **390**, 401-13.
10. Braig, D., Mircheva, M., Sachelaru, I., van der Sluis, E. O., Sturm, L., Beckmann, R. & Koch, H. G. (2011) Signal sequence-independent SRP-SR complex formation at the membrane suggests an alternative targeting pathway within the SRP cycle, *Molecular biology of the cell*. **22**, 2309-23.

11. Wood, H., J, L. & Tollervey, D. (1992) Evolutionary conserved nucleotides within the E.coli 4.5S RNA are required for association with P48 in vitro and for optimal function in vivo, *Nucleic acids research.* **20**, 5919-5925.

12. Wenk, M., Ba, Q., Erichsen, V., MacInnes, K., Wiese, H., Warscheid, B. & Koch, H. G. (2012) A universally conserved ATPase regulates the oxidative stress response in Escherichia coli, *The Journal of biological chemistry.* **287**, 43585-98.

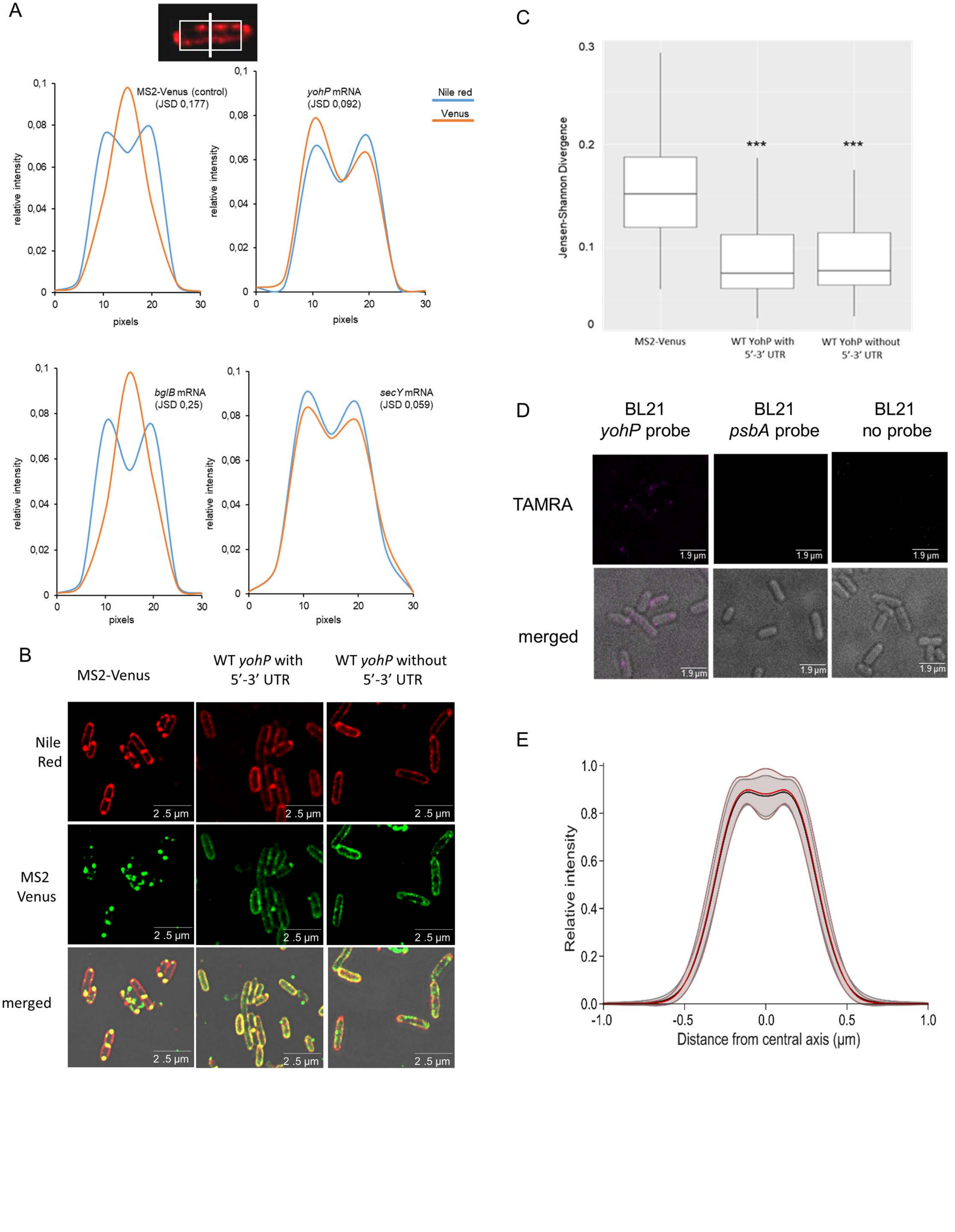

(Sarmah et al., Fig. S1)

A

```
WT-YohP    ATGAAAATTATACTCTGGGCTGTATTGATTATTTTCCTGATTGGGCTACTGGTGGTGACTGGCGTATTTAAGATGATATTTTAA
U-rich     ATGAAAATTATTCTTTGGGCTGTTTTGATTATTTTCCTTATTGGTCTTCTTGTTGTTACTGGTGTTTTTAAGATGATTTTTTAA
C-rich     ATGAAAATCATACTCTGGGCCGTACTGATCATCTTCCTGATCGGGCTACTGGTGGTGACCGGCGTATTCAAGATGATATTCTAA
A-rich     ATGAAAATAATACTATGGGCAGTATTAATAATATTCCTAATAGGACTACTAGTAGTAACAGGAGTATTTAAAATGATATTTTAA
G-rich     ATGAAGATTATACTGTGGGCGGTGTTGATTATTTTCCTGATTGGGCTGCTGGTGGTGACGGGGGTGTTTAAGATGATATTTTAA

U-rich*    ATGAAAATTATACTTTGGGCTGTATTGATTATTTTTCTTATTGGTCTACTGGTGGTGACTGGTGTTTTTAAGATGATTTTTTAA
C-rich*    ATGAAAATCATACTCTGGGCTGTATTGATCATCTTCCTGATCGGCCTACTGGTGGTGACTGGCGTATTCAAGATGATCTTCTAA
A-rich*    ATGAAAATAATACTCTGGGCTGTATTAATTATTTTCCTAATTGGGCTACTGGTAGTAACAGGCGTATTTAAAATGATATTTTAA
G-rich*    ATGAAAATTATACTGTGGGCGGTGTTGATTATTTTCCTGATTGGGCTACTGGTGGTGACTGGCGTGTTTAAGATGATATTTTAA

yohP(Δ4-30)      ATG---------------------------ATTTTCCTGATTGGGCTACTGGTGGTGACTGGCGTATTTAAGATGATATTTTAA
yohP(Δ10-27)     ATGAAAATT------------------ATTATTTTCCTGATTGGGCTACTGGTGGTGACTGGCGTATTTAAGATGATATTTTAA
yohP(Δ46-60)     ATGAAAATTATACTCTGGGCTGTATTGATTATTTTCCTGATTGGG---------------GGCGTATTTAAGATGATATTTTAA

yohP(4-30subs)     ATGAAGATCATTCTGTGGGCAGTTTTAATCATTTTCCTGATTGGGCTACTGGTGGTGACTGGCGTATTTAAGATGATATTTTAA
yohP(10-27subs)    ATGAAAATTACACACTCGGATGCATCGATTATTTTCCTGATTGGGCTACTGGTGGTGACTGGCGTATTTAAGATGATATTTTAA
yohP(46-60subs)    ATGAAAATTATACTCTGGGCTGTATTGATTATTTTCCTGATTGGGCTTCTCGTAGTCACAGGCGTATTTAAGATGATATTTTAA
```

B

Variant	length	A	G	C	T
WT	84	21	20	9	34
U-rich	84	16	15	7	46
C-rich	84	21	20	18	25
A-rich	84	36	13	7	28
G-rich	84	17	28	7	32
U-rich*	84	19	18	6	41
C-rich*	84	20	19	17	28
A-rich*	84	28	15	9	32
G-rich*	84	19	24	8	33
(Δ4-30)	57	13	15	6	23
(Δ10-27)	66	18	15	6	27
(Δ46-60)	69	19	15	6	29
(4-30 subs)	84	20	21	10	33
(10-27 subs)	84	23	19	12	30
(46-60 subs)	84	22	17	11	34

C

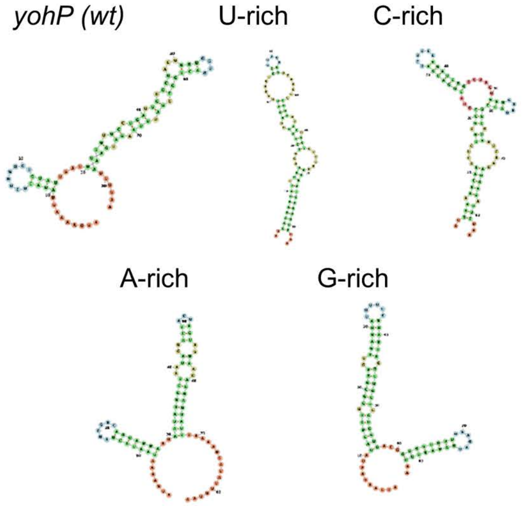

D

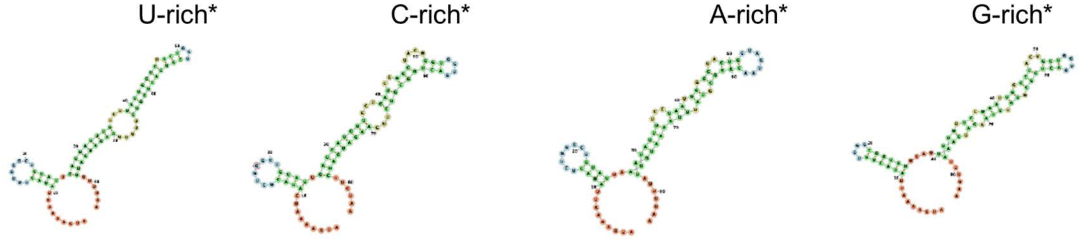

E

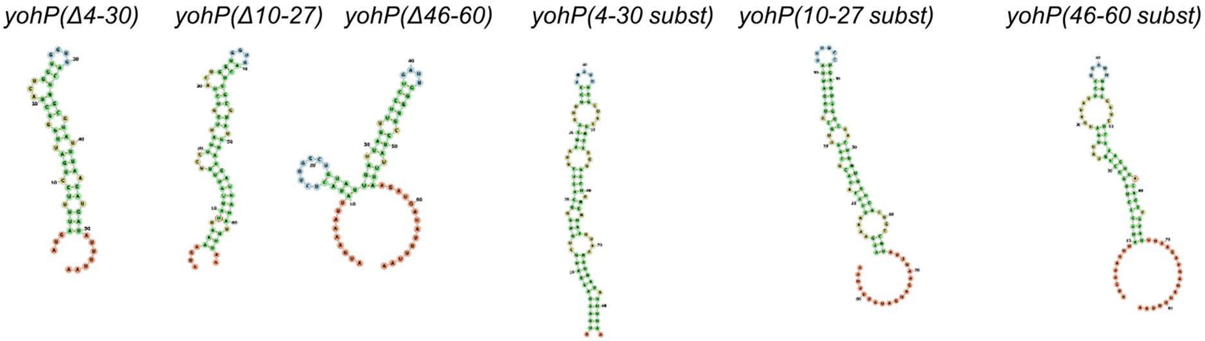

(Sarmah et al., Fig. S2)

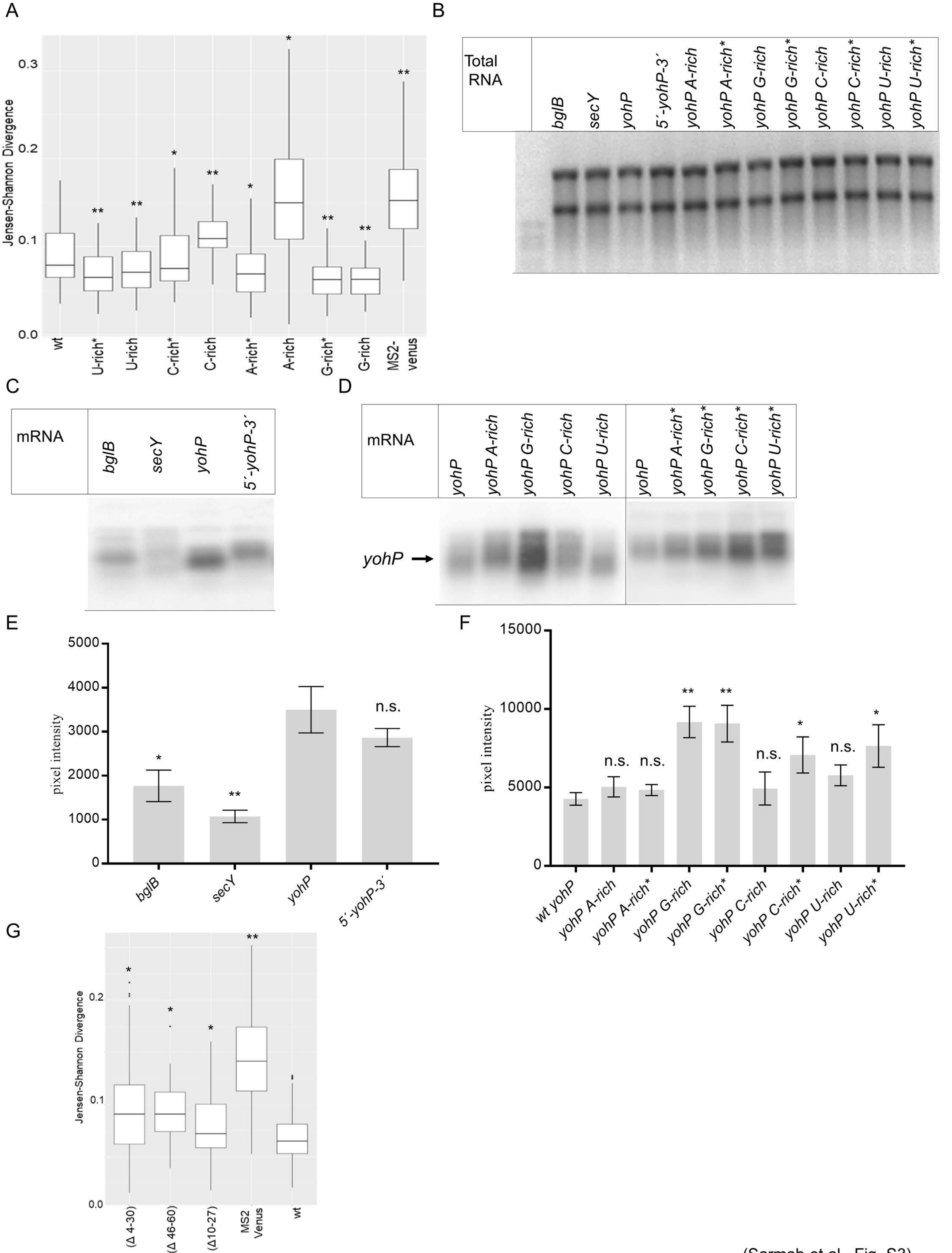

(Sarmah et al., Fig. S3)

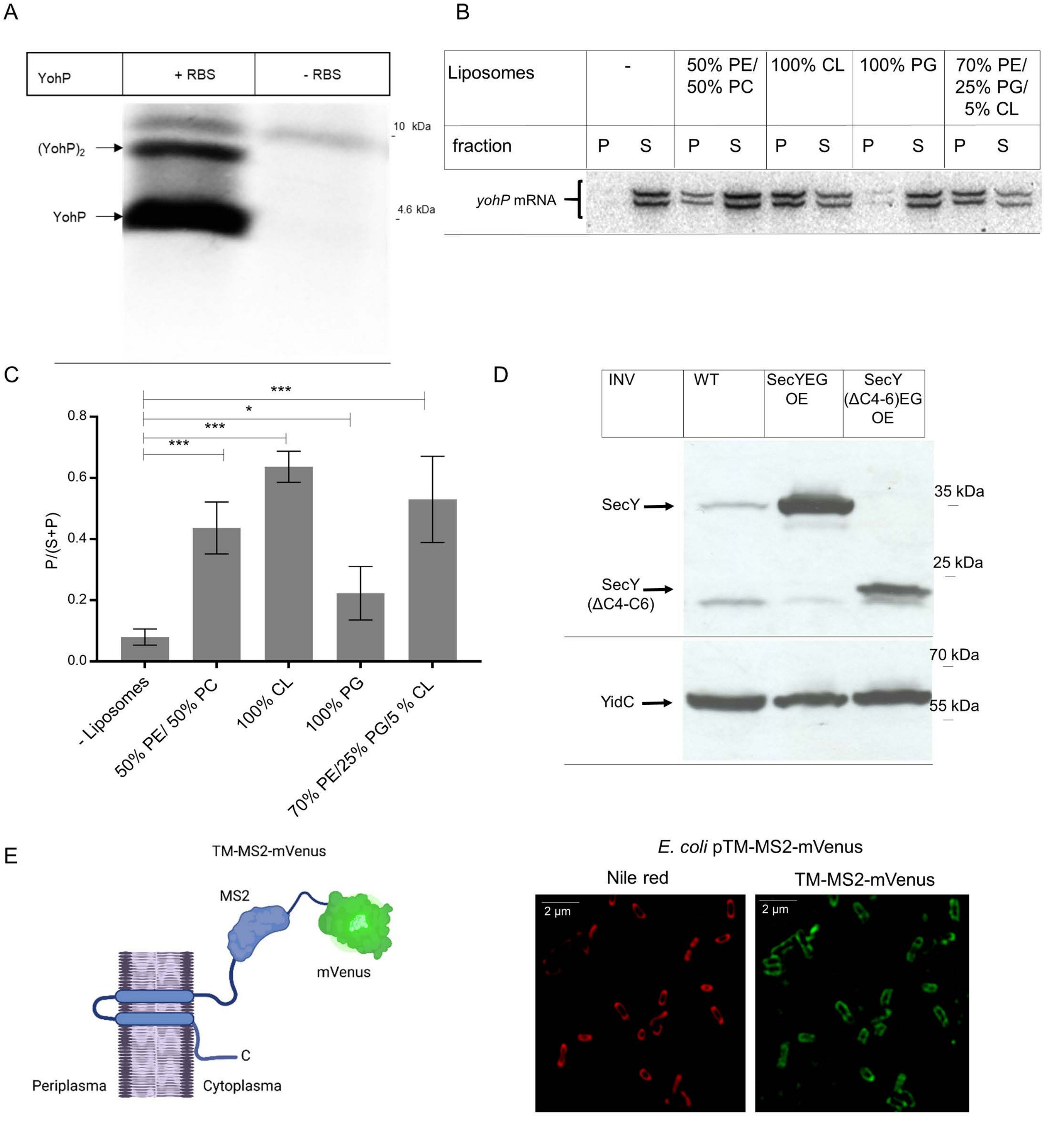

(Sarmah et al., Fig. S4)

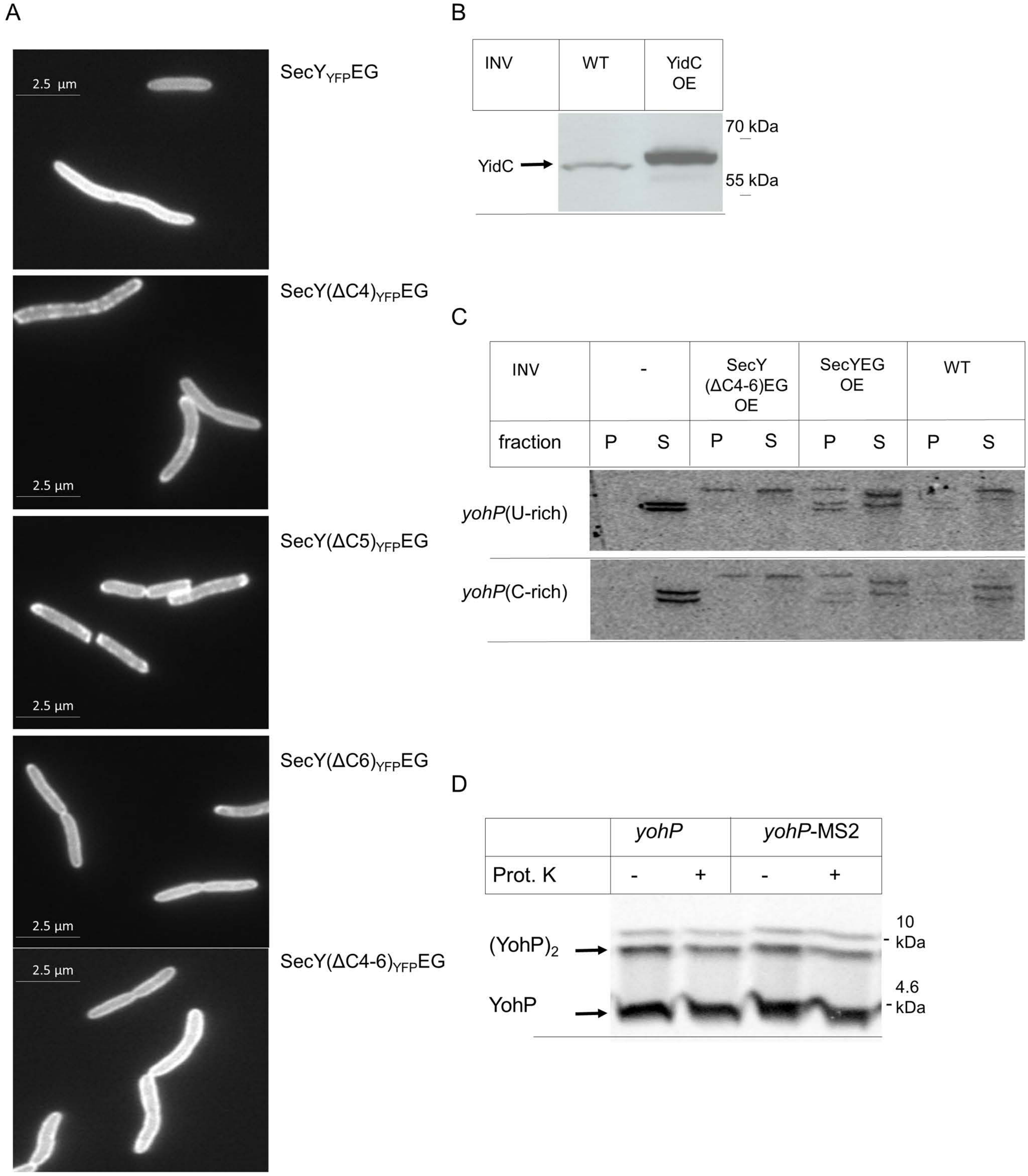

(Sarmah et al., Fig. S5)

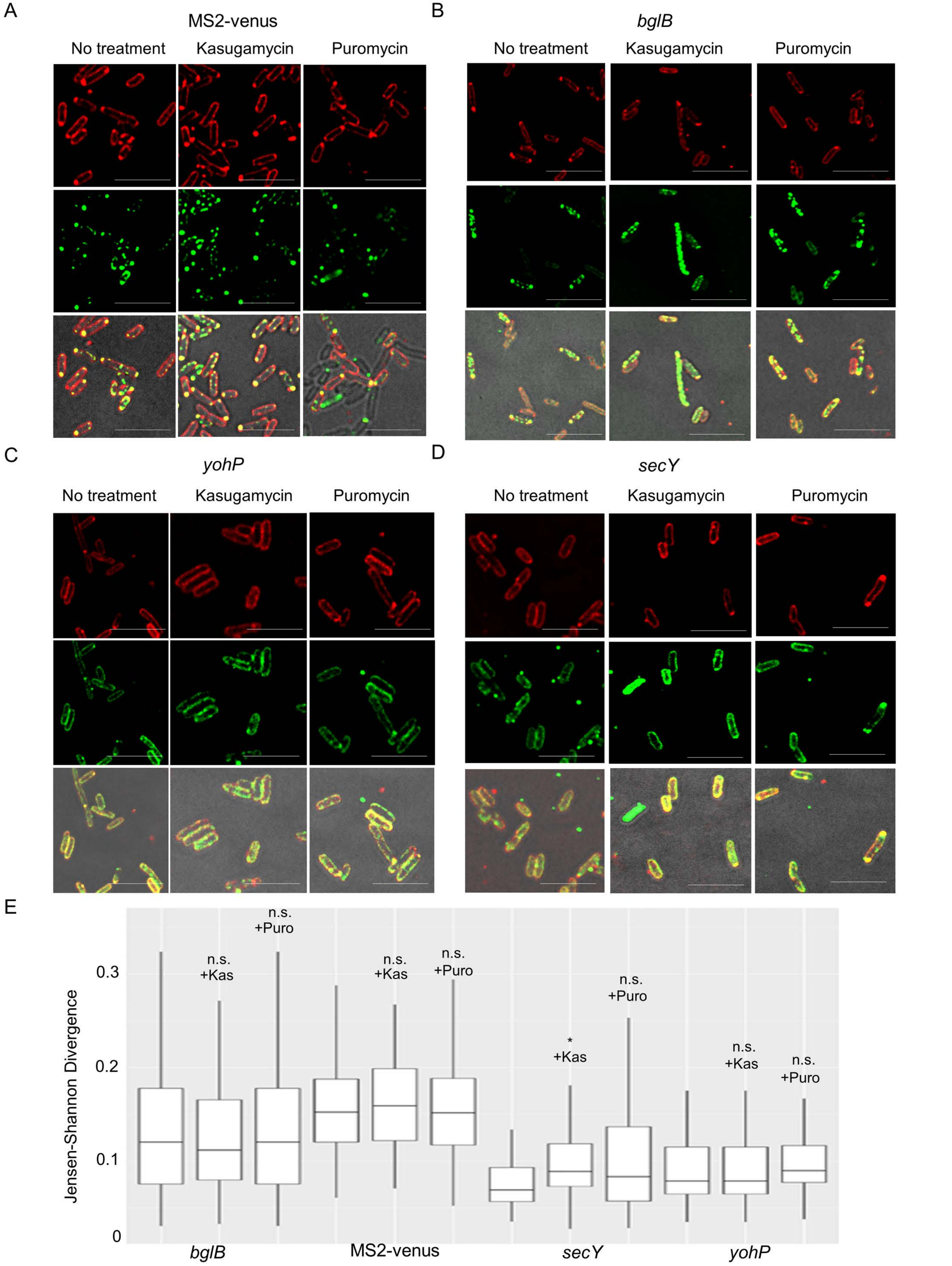

(Sarmah et al., Fig. S6)

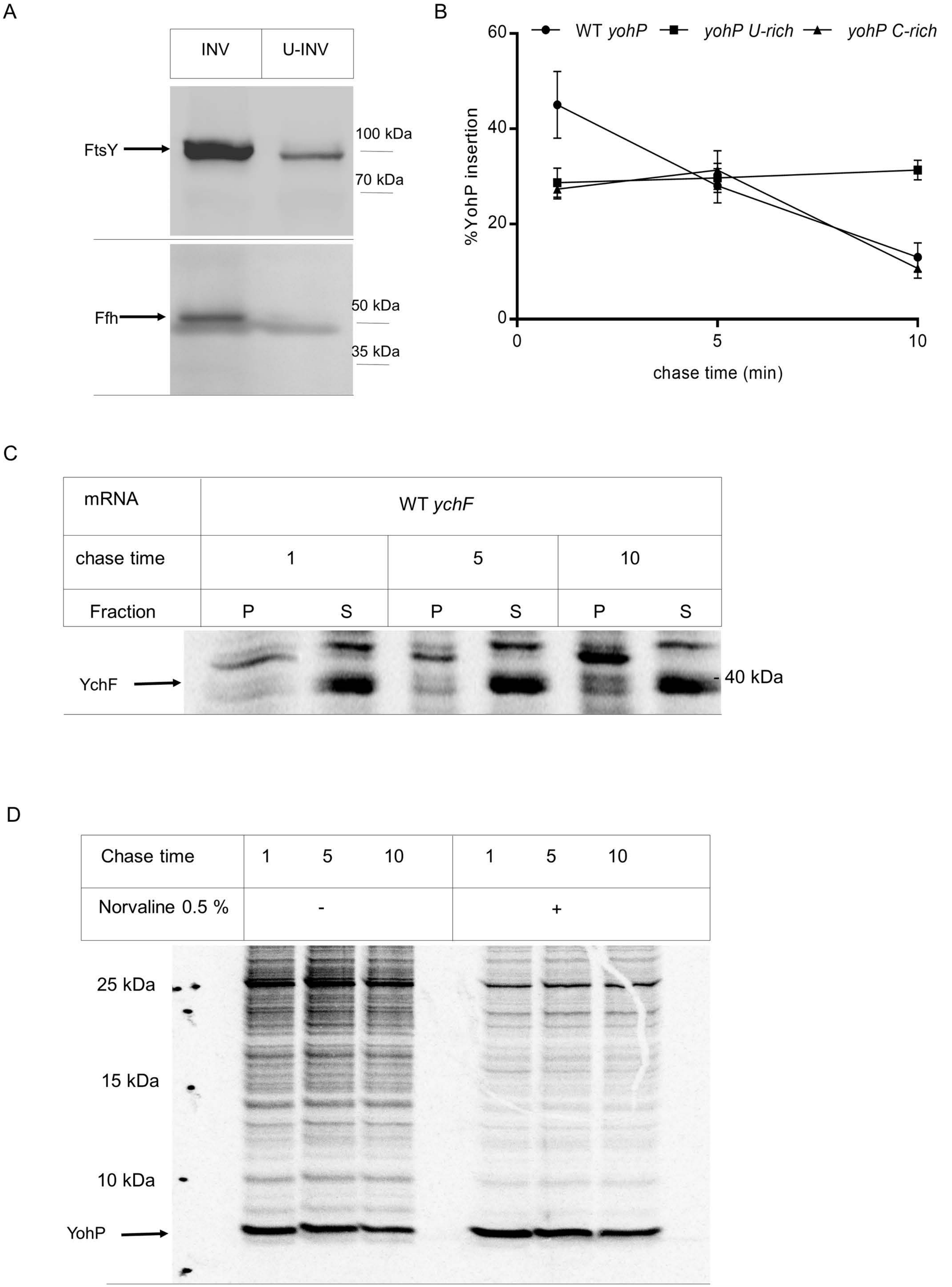

(Sarmah et al., Fig. S7)
